# Carbon Nanotubes and Short Cytosine-Rich Telomeric DNA Oligomeres as Platforms for Controlled Release of Doxorubicin—A Molecular Dynamics Study

**DOI:** 10.3390/ijms21103619

**Published:** 2020-05-20

**Authors:** Pawel Wolski, Krzysztof Nieszporek, Tomasz Panczyk

**Affiliations:** 1 Jerzy Haber Institute of Catalysis and Surface Chemistry, Polish Academy of Sciences, ul. Niezapominajek 8, 30239 Cracow, Poland; pawl.wolski@gmail.com; 2 Department of Chemistry, Maria Curie-Sklodowska University, pl. M. Curie-Sklodowskiej 3, 20031 Lublin, Poland; krzysn@hektor.umcs.lublin.pl

**Keywords:** i-motif, carbon nanotube, drug delivery, doxorubicin, molecular dynamics, force field

## Abstract

This work deals with molecular dynamics analysis of properties of systems composed of carbon nanotubes and short telomeric DNA strands able to fold into i-motif structures at slightly acidic pH conditions. The studies are focused on possible application of such constructs as pH-controlled drug delivery and release systems. We study two different approaches. The first assumes that folding/unfolding property of these DNA strands might realize a gate closing/opening mechanism with carbon nanotube as a container for drug molecules. The second approach assumes that these DNA strands can modulate the drug intercalating property as a function of pH. As a model drug molecule we used doxorubicin. We found that the first approach is impossible to realize because doxorubicin is not effectively locked in the nanotube interior by DNA oligonuceotides. The second approach is more promising though direct drug release was not observed in unbiased molecular dynamics simulations. However, by applying detailed analysis of pair interaction energies, mobilities and potential of mean force we can show that doxorubicin can be released when the DNA strands fold into i-motifs. Carbon nanotube in that latter case acts mainly as a carrier for active phase which is composed of DNA fragments able to fold into noncanonical tetraplexes (i-motif).

## 1. Introduction

The cytosine-rich DNA oligomeres are able to perform reversible structural transitions as a function of pH [1,2,3]. At neutral pH, when all cytosines are in their native nonprotonated forms, the oligomeres form random coils while at slightly acidic pH they can fold into i-motif tetraplexes. Those cytosine-rich oligomeres are naturally present in telomeric regions of chromosomes, centromeres, or in the regulatory region of genes [4].

The controllable folding/unfolding property of i-motifs can be utilized in various areas of sciences and particularly in nanomedicine. Indeed, there were proposed many interesting applications of DNA i-motifs as sensors [5,6,7], nanomachines [8,9,10,11], or drug delivery/controlled release platforms [12,13,14,15,16,17]. Another interesting biomedical aspect of i-motif was indirect inhibition of telomerase activity, as found by Chen et al [18]. That effect involves presence of carboxylated carbon nanotubes which can selectively induce i-motif formation also at neutral pH and facilitate G-quadruplex formation in the complementary dsDNA strand [18,19,20,21].

Telomeric sequences of cytosine-rich strands were quite extensively studied as pH-controlled carriers of drugs. The most frequently studied drug molecule seems to be doxorubicin, DOX. It is a well-known anticancer drug with confirmed efficacy but also with confirmed severe side effects. Therefore, i-motif and DOX were often used as a model system designed to immobilize or inactivate DOX in some way at neutral pH but releasing it at acidic pH of tumor microenvironment. DOX was intercalated in the DNA duplex at neutral pH. The duplex was formed by a sequence able to fold into i-motif and the other complementary strand. Usually, one of these DNA strands was attached to some carrier and the second was allowed to form Watson-Crick pairs with the first one. Then intercalation of DOX was performed at neutral pH. At acidic pH, according to published data, the duplex disassociated forming the i-motif and DOX was then released [14,15,16,17]. Various concepts were used to immobilize i-motifs to form a working carrier, among the others gold nanoparticles [14,17] exomes [16] or mesoporous silica nanoparticles were studied for that purpose [12,13]. Also, nanocomposite materials based on silica nanoparticles, carbon nanotubes and DNA fragments were studied as materials for controlled release of DOX [22].

A common point of all the above-mentioned studies was the lack of a closer theoretical (or in silico) studies confirming the assumed working schemes. Of course, experimental verifications were usually provided and this is strong proof of concept. However, sometimes the proposed mechanisms of actions were very complex and involved very subtle changes in binding energies being a result of actually small changes in pH. For example, according to DOX intercalation mechanism, it was usually assumed that Hoogsteen pairing, within the semiprotonated cytosines pairs, in i-motif prevailed over the Watson-Crick pairing. As a result, one of the strands forming the duplex had to be detached and rejected in favor of the formation of i-motif at acidic pH. As a result, the previously intercalated DOX could be released to the bulk. Therefore, the question of whether it is the only possible mechanism or one among a few is difficult to answer and an in silico analysis would bring useful complementary information in these circumstances.

This work is thus an attempt to fill the gap in methodology applied to study drugs releasing feature of i-motif containing constructs. Of course, we are not able to analyze each of the studied cases. Instead, we focus on one of the simplest and most common cases that are constructs formed by telomeric DNA sequences and carbon nanotubes. That choice is justified by quite intense studies related to application of carbon nanotubes as drugs carriers [23,24,25] and also to the reported specific interaction of CNTs with DNA motifs [5,6,18,21] As a model drug we still use doxorubicin, partially due to already mentioned reasons, but also due to already confirmed ability to control DOX release from carbon nanotubes by pH change of the environment [23,24,26].

We first analyze a gate opening/closing mechanism by cytosine-rich telomeric DNA sequences and carbon nanotube as DOX storage platform. In the next step, we analyze coadsorption of cytosine rich sequences and DOX on CNT sidewalls and check the ability of DOX immobilization and release due to formation of i-motif structure at acidic pH. Finally, we focus on the closer analysis of the interaction sites of DOX on i-motif and determine potential of mean force associated with the adsorption of DOX on these cytosine-rich DNA fragments.

## 2. Results and Discussion

### 2.1. Definitions of the Analyzed Systems and Computational Details

The studies were carried out by combining a few of four different components in a single simulation box. Each of these four components was build (geometry and its force field) according to its specific needs and also intermolecular interactions between these components had to be set up accordingly.

**Figure 1:**
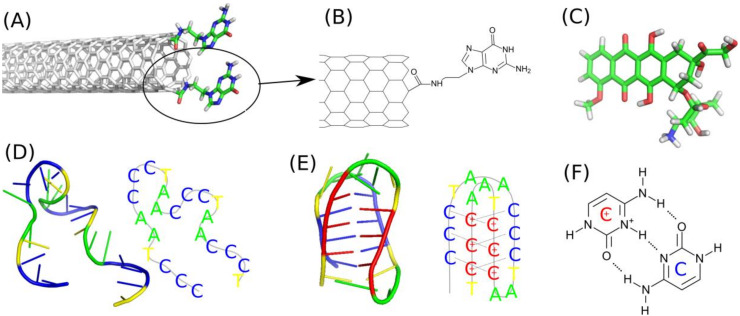
(**A**) Illustration of the applied functionalization of (10,0) carbon nanotube. (**B**) Schematic representation of the molecular topology of the guanine functional group attached to the CNT tip. (**C**) Doxorubicin molecule in its protonated form. (**D**) iMu - the telomeric fragment of ssDNA consisting of the sequence CCCTAACCCTAACCCTAACCCT in the radom coil form. (**E**) iMp - the fragment of ssDNA with semiprotonated cytosines CCCTAACCCTAAC^+^C^+^C^+^TAAC^+^C^+^C^+^T in the form of i-motif. (**F**) Triplet of hydrogen bonds formed between protonated C^+^ and unprotonated cytosines C within the iMp structure.

The first component is the functionalized carbon nanotube fCNT. We build the (n,m) carbon nanotubes by using self-designed script. Two types of carbon nanotubes we studied: (10,0) and (20,0). The diameters of that zigzac CNTs are shown in Table 1 and they can be easily calculated from their chiral index as 0.78 times n. The nanotubes were covalently on-tip functionalized by guanine, Gu, containing residues as shown in Figure 1A,B. That kind of functionalization was described in literature [27] and involves carboxylation and subsequent derivatization with N’9-(2-aminoethyl) guanine. Preparation methodology of the molecular model of the functionalized nanotube was the same as already described in one of our recent publications [28]. Shortly, we first prepared a template of the guanine containing residue with only one aromatic ring cut off the CNT structure. That template was subjected to quantum chemical calculations in order to determine the values of point charges. To that purpose, we took advantage of the RESP/ESP charge derive server [29] (https://upjv.q4md-forcefieldtools.org/REDServer-Development) and next we build the force field topology according to the generalized amber force field, gaff, using AcPyPE script [30,31,32]. In the next step we manually grafted the Gu residue into the CNT geometry remembering about new bonds, angles, and dihedrals formed with the carbon atoms belonging to the CNT.

The internal structure of CNT was described using the AIREBO force field [33,34]. This a reactive empirical potential which correctly reproduces mechanical properties of carbon nanotubes but in this case its reactive property was actually not used. Interaction of that functionalized carbon nanotube with other atoms present in the system was described classically according to Lennard-Jones 12–6 potential and Coulomb potential when a given pair of atoms had nonzero point charges. Carbon atoms belonging to the CNT core were treated as classical aromatic sp2 carbons with LJ parameters taken from gaff force field (‘ca’ atom type). Standard Lorentz-Berthelot mixing rules were applied in the case of mixed interactions between atoms from fCNT and, for example, atoms belonging to doxorubicin molecule. 

Doxorubicin, DOX, was used in its protonated form (Figure 1C) because in the considered conditions of pH (5–7) and its pK_a_ = 8.2 the standard unprotonated form is very unlikely [35] The force field components for DOX were determined according to the same scheme like in the case of Gu residue. Thus, first the partial charges were determined using RESP scheme and next the full force field topology was generated using AcPyPE script which calls various routines from AmberTools package.

The two other components were nucleic acid sequences in a form of single stranded DNA fragments representative to telomeric region of chromosome. Precisely, these are only the cytosine-rich strands which are able to make reversible transitions from random coil form (Figure 1D) to the i-motif form (Figure 1E) depending on pH or protonation state of cytosines. The structure in Figure 1D is the representative structure at neutral pH when all cytosines are in their native unprotonated forms. We will call those ssDNA sequences as iMu because they use unprotonated cytosines. The structure from Figure 1E is the representative one at acidic pH when the sequence folds into noncanonical i-motif structure which is kept by triplets of hydrogen bonds (Figure 1F). We will call this state as iMp as it contains protonated cytosines. Both iMu and iMp will be sometimes called as iM when their symmetry or protonation state is not important. 

**Figure 2:**
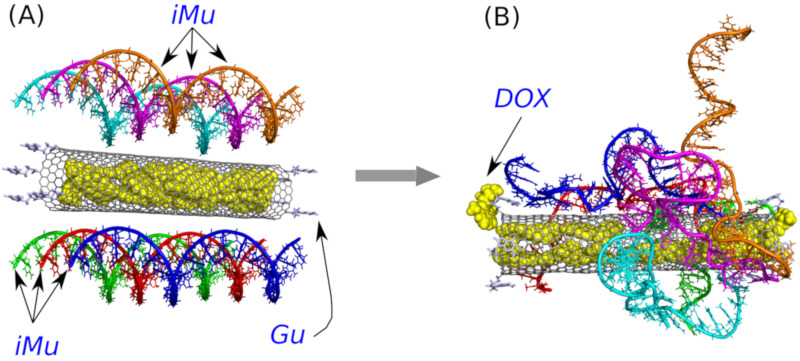
(**A**) The initial structure with doxorubicin (DOX) encapsulated in the CNT interior and six unprotonated iM sequences, iMu, placed in the vicinity of the CNT surface. For a better clarity, each iMu is drawn in different color though all of them are identical. Carbon nanotube is on-tip functionalized by guanine, Gu, residues. (**B**) The same structure after 40 ns of simulations.

The iMu atomic structure was build using a script which generates ideal ssDNA strand (see Figure 2A for illustration). The force field associated with that structure was build using the tleap program from AmberTools package with the included calls to ff99 force field for nucleic acids including the bsc1 modifications [31,36]. The iMp structure was however produced using the pdb file (pdb id 1EL2) published by Phan et al. [1,37] with the sequence CCCTAA5mCCCTAAC^+^C^+^C^+^UAAC^+^C^+^C^+^T folded into i-motif. We replaced the 5mC and U residues by standard cytosine and thymine in order to obtain the true human telomeric i-motif molecules. Furthermore, the force field components were generated using the same scheme as in the case of iMu but here we additionally added the libraries for protonated nucleic acids in tleap script.

As already mentioned, the components described above were picked up and put into the simulation box in various combinations using self designed scripts. The interaction between atoms belonging to individual components was calculated using the standard non-bonded pair potentials (Lennard-Jones 12–6 and Coulomb with summation in reciprocal space). Obviously, the same non-bonded pair potentials were used for the interaction with water molecules and ions. The applied water model was the standard TIP3P with shake algorithm for making water molecules rigid. The Na^+^ and Cl^−^ ions were added in amounts necessary to make the whole system charge neutral and to produce 0.145 mol L^−1^ ionic strength of solution. The Lennard-Jones parameters of these ions were taken from the amber force field for nucleic acids. The number of water molecules varied depending on the system composition, but it was always close to 31000 and the initial simulation box dimension was 100 × 100 × 100 Å^3^. 

The calculations were carried out using LAMMPS [38] molecular dynamics engine with the integration timestep 1.8fs and periodic boundary conditions applied in all directions. The pair interaction cutoff was 12 Å for both Lennard-Jones and Coulomb potentials computed in real space. The summation of electrostatic interactions in the reciprocal space was done according to particle-particle particle-mesh solver, as implemented in LAMMPS. The pressure and temperature were controlled using the Nose-Hoover algorithm. 

The calculations were essentially divided into two parts. The first part was devoted to search for the thermodynamically optimal configuration. The second stage was the standard production run carried out at NPT ensemble. Some systems were subjected to additional calculations aimed at determining the potentials of mean force associated with some specific transitions. The most critical stage of calculation was the first one associated with the equilibration run. The considered systems are large and are composed of strongly interacting big macromolecules and some of them reveal a fragile internal structure. Thus, in order to find optimal configurations in a relatively short time and without destroying the internal atomic structures of i-motifs we applied the recently developed rigid body replica exchange scheme [39]. Without going into details that scheme consists of repetitive sequences of runs where the system is switched into implicit solvent and its essential components are subjected to rigid body dynamics and next the resulting most energetically favorable state is subjected to relaxation in standard explicit solvent run. These sequences are repeated until the resulting configuration does not change significantly. In the current cases, the number of successive stages of rigid body and relaxation runs was 3 which corresponded to ca. 48 ns of real time for each system. The production runs took from 50 ns to 72 ns depending on the system size while the runs devoted to determination of potentials of means force took additionally ca. 40 ns.

The studies were carried out for three separate cases focusing on different molecular architectures and research problems. These cases are formally called as A, B, and C and subsequent numbers denote different compositions within a given case. Table 1 provides all the details concerning systems types and compositions.

The case A was studied in order to check whether the unprotonated i-motif iMu is able to wrap the nanotube and block its open ends against DOX release. It is comprised of a wide (20,0) nanotube, which is able to store DOX molecules encapsulated in its interior, and 6 iMu molecules adsorbed on the sidewall of the nanotube. 

The case B consists of four subsystems differing in protonation state of cytosines and number of DOX molecules. All nanotubes in this case are (10,0), i.e., narrow nanotubes unable to store DOX or anything else in their inner spaces. Either iM or DOX are adsorbed on the external sidewalls of the nanotubes.

In the case C we focus on the interaction of iM with DOX without the presence of carbon nanotube. We consider different protonation states of iM and various numbers of DOX molecules.

### 2.2. I-Motif as a Gate Closing/Opening Factor

The well-known property of iM, that is ability to perform reversible folding/unfolding transitions [3,40,41] encouraged us to study application of iM as pH-controlled cap of open ended carbon nanotube. We hypothesized that unfolded iMu can block the release of drug molecules (doxorubicin) encapsulated previously in the nanotube inner space. This hypothesis was supported by the observation that unfolded iMu is highly flexible and may tend to wrap the CNT surface and partially incorporate to its interior. Similar mechanism of blocking the CNT interior has already been observed in our recent studies related to blocking of CNT by nanoparticles and polyethylene glycol chains adsorbed on the CNT surface [42]. In this case, we assumed that unprotonated and unfolded DNA sequence will form a bundle, blocking the CNT interior, due to interaction with guanine residues. This bundle should however disappear at slightly acidic pH when the spatial stiff structure of iMp forms and the CNT interior should become unblocked. 

Because the key factor is the ‘gate closing’ ability of the unprotonated iMu we started the analysis from such a case. Figure 2 shows two structures with the unprotonated iM, that is iMu: the initial one and the structure which formed spontaneously after 40 ns of simulations. As seen doxorubicin was initially placed inside the nanotube and the nanotube was surrounded by 6 iMu chains. This architecture was subjected to equilibration and normal runs with water and ions in NPT ensamble. DOX was initially blocked by non-permeable walls localized at CNT tips to prevent their escape at very beginning. However, after a few nanoseconds the walls were removed and the system evolved without any bias.

Analysis of the system configuration in Figure 2B leads to simple conclusion. Namely, the iMu chains grouped together without any tendency to occupy the CNT tips and form bundles. As a result, the CNT entrances are open and DOX can spontaneously escape from the interior of (20,0) nanotube. Indeed, we observed two DOX molecules beyond the CNT walls after 40 ns of simulations and obviously all of them would escape from the CNT in the limit of macroscopic time. This is because the energetic barrier against DOX release from (20,0) nanotube is ca. 22 kJ mol^−1^, i.e., the value which can easily be surmounted by thermal energy [24]. Therefore, we postponed further analysis of that system and the concept of gate closing/opening by folding/unfolding of iM in response to pH change has been postponed either.

### 2.3. Co-Adsorption of I-Motif and Doxorubicin on the Surface of Carbon Nanotube

Another molecular architecture involving iM, DOX, and CNT is their mutual interaction leading to co-adsorption of iM and DOX on the CNT external surface. Four different cases were subjected to careful analysis and they are defined in Table 1 as cases B1–B4. The joint element of these four cases is the narrow (10,0) nanotube which tips were functionalized by guanine residues, Gu. This kind of functionalization was already studied by us and we found that Gu can enhance the interaction of iM alone [28] or being a part of a larger structure [39] when compared to a non functionalized nanotube. The carbon nanotube plays in this case rather the role of a scaffold for iM and DOX since the internal space of (10,0) cannot store any external species due to its small diameter. 

**Figure 3:**
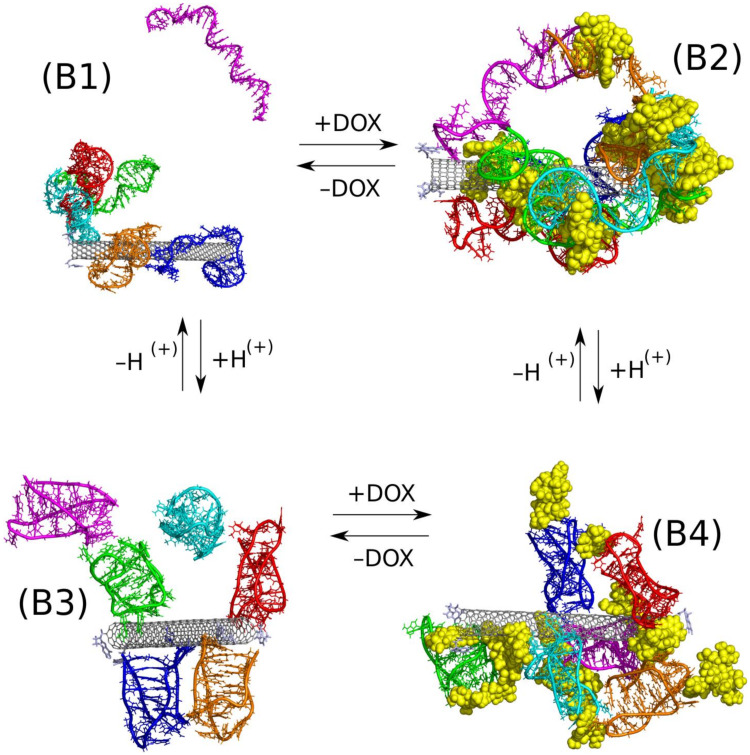
Optimal configurations of the systems B1–B4 found according to the scheme of rigid body replica exchange method followed by ca. 50 ns of unbiased calculations in NPT ensemble. Each iM molecule is displayed in different color in order to make the presentation clearer, DOX is shown as yellow spheres. The +/− DOX and +/− H(+) arrows inform which change of the system state is done, i.e., incorporation/removal of DOX, application of protonated/unprotonated iM.

The structures composed of three relatively large species and one of them additionally able to make internal transitions require special attention during calculations. It is obvious that we need to reach configuration close to thermodynamic equilibrium however simple unbiased simulations lead normally to ‘glassy’ states, blocked at quite random configurations due to large energetic barriers against any displacement. On the other hand, application of typical biasing methods may lead (and normally leads) to destruction of the internal architecture of iM, particulary its iMp symmetry. Therefore, in order to find thermodynamically optimal configurations, we applied the recently developed methodology which helps to keep internal structure of selected objects intact and, at the same time, allows for intense probing of the intermolecular configurations. That methodology is based on periodic changes of the system type and interactions topologies [39]. Shortly, it consists of repetitive stages of calculations assuming parallel tempering of the rigid body motions at implicit solvent and unbiased all-atom calculations at explicit solvent. In this case the rigid body/all-atom stages were repeated three times and it was enough to obtain configurations which produced the strongest interaction between the essential system components. The optimal configurations of the mixed systems fCNT + iM + DOX are presented in Figure 3 and mean pair interaction energies between these compounds are collected in Table 2.

Let us first consider how protonation state of iM affect the adsorption of iM on the fCNT surface without DOX. We assume that in the context of the drug delivery the representative structure of iMu is fully unfolded state (circulation of the carrier in the blood stream at pH = 7.4). The representative structure of the protonated iMp is, in turn, when the carrier enters the acidic microenvironment of the tumor site. Thus, looking at the switch between B1 and B3 case we can notice significant structural differences between these two cases. In B1, the iMu is in the form of random coil and can easily wrap the nanotube surface and indeed two iMu molecules did it. The others formed a cluster which attached to one iMu molecule adsorbed on the CNT surface. In this case, the interaction energy of iMu with fCNT is the highest and also pair energy between iMu molecules is one of the biggest (electrostatic part dominates). However, we cannot consider that structure as stable because one of iMu molecules spontaneously detached from the cluster and went to the bulk. The direct interaction and wrapping of fCNT by iMu probably leads to a very stable construct but attachment of other iMu molecules to already adsorbed ones is rather weak. 

The protonated iMp, case B3, form rather loosely structures either with fCNT or with other iMp. We can thus conclude that transition from random coil (iMu) to true i-motif structure (iMp) leads to weakening of the interaction with carbon nanotube. This is also confirmed by the pair interaction energies in Table 2. They drop significantly when going from iMu to iMp.

An interesting conclusion came from the observation of numbers of clusters formed in the above two cases. Prior to their analysis, let us quickly discuss how the clusters are defined and calculated. We assumed that a given molecule/object belong to a single cluster when the distance between at least two atoms is smaller than 3.5 Å. However, we intentionally neglected atoms belonging to fCNT and DOX in cluster analysis because the most interesting is iM molecules arrangement. Thus, in the case of B1 there are two clusters and they are simply formed by one iMu molecule which detached from the others and the second cluster is formed by the remaining molecules. In the B3 case, we counted four or five clusters within the considered period of time. Thus, the conclusion is that protonated iMp molecules do not tend to group into bigger structures but prefer to move on the fCNT surface individually. Contrary to that, the iMu molecules prefer formation of bigger structures which adsorb on the fCNT surface collectively and tend to wrap the fCNT surface. Thus, the above observation suggests that at neutral pH the fCNT/iMu system may lock other molecules within its structure and unlock at acidic pH when the fCNT/iMp structure becomes loosely.

Let thus analyze such a mechanism using DOX as guest molecules and fCNT/iM as a pH switchable matrix. Thus, the transition from B2 case to B4 case is the example of the mentioned mechanism. As seen in Figure 3 the differences between B2 and B4 are rather small and we do not observe any spectacular effects. In both cases, we obtained constructs comprising of all species present in the simulation box and particularly we do not observe release of DOX in the case of protonated iMp, that is in the B4 case.

A closer analysis of B2 and B4 systems leads however to quite intriguing conclusions. It is seen that in the B2 case DOX molecules are entangled in the network of iMu molecules which, in turn, are firmly attached to the fCNT surface. iMu molecules form a single cluster which means that all of them are connected by at least one pair of atoms. In the B4 case, the iMp molecules are grouped into four individual clusters without any contact between them and DOX molecules form bridges between iMp molecules and it seems that they are more exposed to the solvent. It is confirmed by the SASA values calculated for DOX molecules for both cases. Simply, at neutral pH DOX molecules are well surrounded by iMu molecules while at the acidic pH they become more exposed to the bulk. Weaker interaction of DOX with fCNT is also confirmed by the pair interaction energies collected in Table 2. Also, interaction of DOX with iM molecules is weaker in the case of B4 system. All that means that there is a high chance that DOX molecules can detach easily from the carrier at acidic pH or at least they can interact with other species due to a better exposition to the bulk. 

Another interesting observation is that DOX is a kind of stabilizing factor of either iMu or iMp on the fCNT. As seen the lack of DOX molecules (cases B1 and B3) leads to detachment of at least one iM molecule from the cluster formed by the other species. Thus, iM molecules are not strongly bound to the fCNT though pair interaction energies in Table 2 seem to be higher than in relevant cases with DOX present in the system. However, DOX act as bridges between iM molecules (high pair energies between iM and DOX) and thus makes the whole structure stiffer. Incorporation of DOX (cases B2 and B4) leads to formation of a one macromolecule with strong intramolecular interactions which prevent individual iM molecules from the detachment. A similar effect, agglomeration of normally well soluble molecules due to the presence of DOX, was observed in the case of various bisazo dye solutions though those systems belong to totally different class of systems [43]

The role of DOX in the behavior of B2 and B4 systems is even more intriguing if we look at the RMSD plots for these systems. These plots were calculated from the last 10.8ns of simulations using as the reference state the first frame from that period. The RMSD were calculated by subtracting the motions of the centers of mass and making optimal rotations in reference to the first frame. The RMSD plots were prepared in three styles; the first is obtained by taking into account all atoms in the calculation of RMSD, the second style corresponds to the situation when the atoms belonging to DOX molecules were neglected in the computation while the third style assumes that atoms belonging to iM were neglected in the computations of the RMSD. 

**Figure 4:**
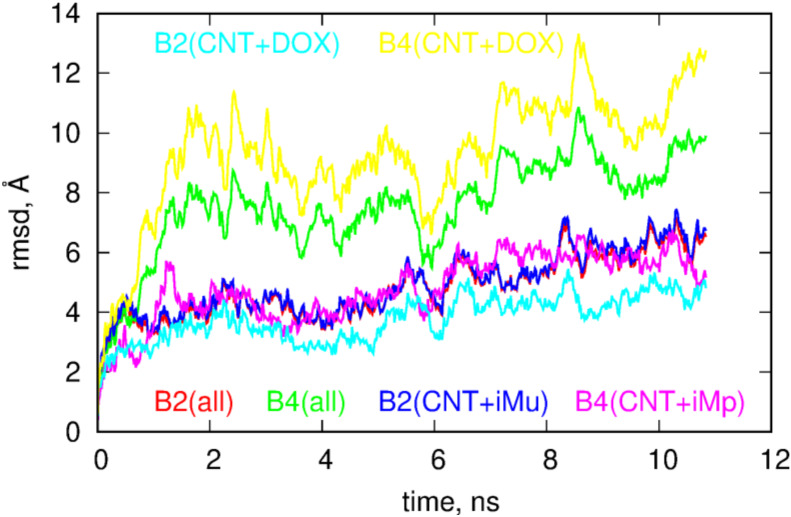
Plots of root of mean squared displacements, RMSD, as a function of time for systems from Figure 3. The label ‘all’ means that all atoms were taken into account in the computation of the RMSD, ‘CNT+iMu/p’ means that atoms belonging to DOX molecules were not accounted for in the computation and ‘CNT+DOX’ means that only atoms belonging to CNT and DOX were accounted for in the computation of the RMSD.

Looking at Figure 4 we notice that both systems reveal structural changes in time but the B4 case definitely performs more intense motion than the B2 case. Analysis of the RMSD plots with DOX or iM subtracted leads to the conclusion that DOX in the B4 systems is much more mobile than in the B2 case and higher values of total RMSD in B4 are governed by DOX motion in this case. Removal of atoms belonging to DOX leads to flattening of RMSD in B4 case and it becomes similar to the B2 case. Thus, having in mind that in B4 case DOX is more exposed to the bulk, moves faster and reveals smaller pair interaction energy with fCNT we can expect that DOX molecules can be easily detached from the whole structure at acidic pH, i.e., in the B4 case. That conclusion can be supported by the analysis of the free energy of binding but due to the system size, complex geometry and composition it would be very difficult to obtain reliable results. However, remembering that binding of DOX is done mainly through iM molecules (not through direct interaction with fCNT) it would be beneficial to perform closer analysis of such a simplified system and this is the subject of the next section.

### 2.4. Interaction of Doxorubicin with iMu and iMp

**Figure 5:**
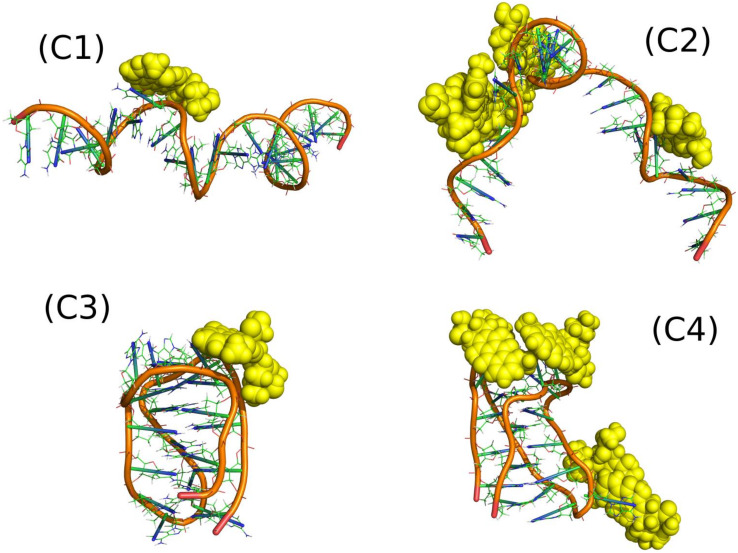
Optimal configurations found for (C1) one DOX molecule interacting with iMu; (C2) four DOX molecules interacting with iMu; (C3) one DOX molecule interacting with iMp; and (C4) four DOX molecules interacting with iMp.

The systems denoted as C1–C4 in Table 1 represent very simple cases showing how DOX interacts with single iM molecules. Figure 5 shows the relevant snapshots from the simulations representing the thermodynamically optimal configurations while Table 3 shows mean pair energies determined between various systems components.

The systems C1 and C3, with only one DOX molecule, were studied in order to identify the strongest binding sites of iM. It was fairly easy in the case of iMp (C3) as the iMp structure is very stiff but rather difficult in the case of iMu. We can notice that the spatial structure of iMu changed significantly when more DOX molecules interacted with it. C1 case reveal smaller energy than C3 and much smaller than C2. Thus, we can conclude that iMu changes its structure depending on the number of DOX molecules interacting with it and also the mean interaction energy changes accordingly. Four DOX molecules led to significant deformation of iMu from almost straight chain into a strongly curved, almost hairpin form. This is associated with a strong pair energy change: from weak (C1) to very strong (C2). Thus, many DOX molecules develop strong interaction sites on the iMu but the switch from iMu to iMp leads to the weakest adsorption of a single DOX molecule among the considered cases. This is important conclusion in the context of DOX release from constructs build of iM molecules due to pH change. 

The iMp has however strong interaction site because the single DOX molecule reveals the strongest pair interaction energy for C3 system. At the same time other areas of iMp must interact with other DOX molecules very weakly since the energy calculated per single DOX molecule in the C4 case is small. The above conclusion must however be verified by the free energy analysis since pair energies are not ultimate descriptors of the binding strength.

Interaction energies of DOX and iM with water support the conclusions drawn from visual analysis of snapshots in Figure 5. The most open iM structure is in the case C1 and it reveals the highest interaction energy with water. It gradually decreases when going from C1 to C4 and it is accompanied by more and more compact structure of iM and ‘screening’ of iM surface by DOX molecules. Pair interaction energy of DOX with water brings another portion of useful information. It is seen that at neutral pH DOX molecules are somehow isolated from water by iMu structure, but at acidic pH they become more exposed to solvent due to switch of iMu into iMp.

**Figure 6:**
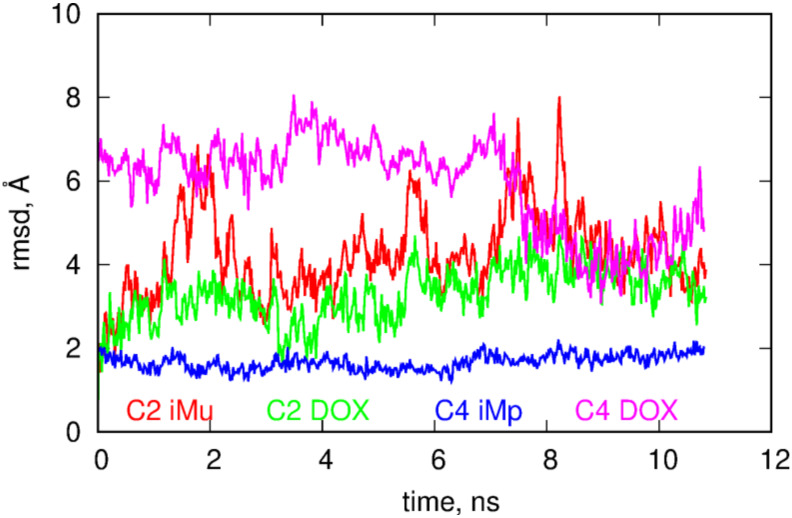
RMSD for components of the C2 and C4 systems. As the reference structure the states obtained in the previous 10.8 ns were used.

The more open iMu structure is accompanied by quite intense changes of its internal atomic configurations. It is seen in Figure 6 where the RMSD plots of components of C2 and C4 systems are shown. It is obvious that iMu reveals structural changes continuously but also DOX in the C2 seems to perform intense internal motion. However, DOX simply follows the motion of iMu because the RMSD curves for both compounds are highly correlated. Totally different behavior is observed in the C4 case. The iMp is totally static but DOX reveals very intense motion. This confirms the already drawn conclusion that DOX release from the iMp is easier than from the iMu.

**Figure 7:**
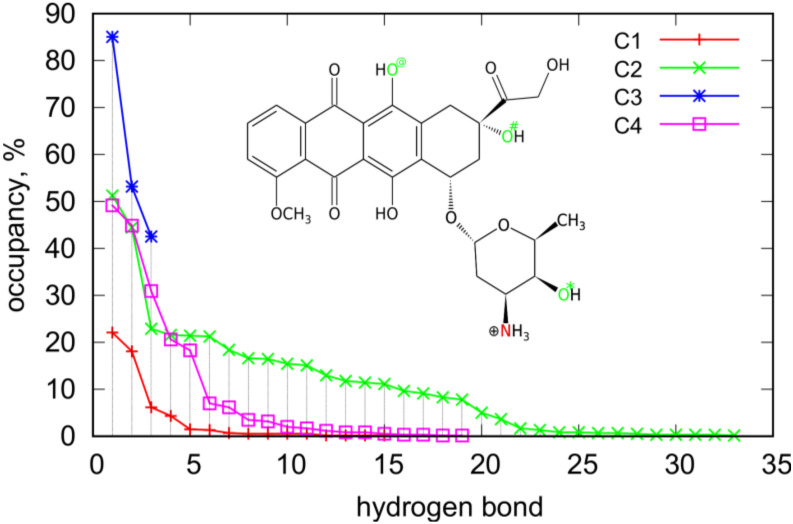
Occurrence and number of hydrogen bonds between DOX and iM observed during the runs for C1–C4 systems. The nitrogen and oxygen atoms in the structural formula of DOX show the essential interaction sites of DOX with iM.

Closer analysis of the interaction of DOX molecules with iM allows us to detect the most essential interaction sites. The analysis was based on the detection of hydrogen bonds formed because they normally have a dominant contribution to the non-bonded energy between two individual compounds. Table 3 shows how many H-bonds were observed during the runs in the C1–C4 systems and Figure 7 presents how the occurrence of a particular bond was during the run. 

The total number of H-bonds per single DOX molecule informs on the extent of strong interaction areas on the iM surface. These values were directly determined from the simulation snapshots using standard criteria of distance and angle between donor and acceptor atoms (less than 3 Å and 20 deg). In the case of systems C1 and C3 they were counted directly but in the case of C2 and C4 the total number of H-bonds was divided by four (values in parentheses in Table 3). Therefore, looking at the values in Table 3 we can conclude that unfolded iMu is much richer in strong interaction sites than the folded iMp. This effect is due to more open iMu structure and a better availability of atoms able to form H-bonds with DOX molecule. 

An interesting case is C3 where only 3 H-bonds were observed and at the same time their occurrence/occupancy is the highest, as shown in Figure 7. This is correlated with the highest pair interaction energy between DOX and iMp. In this case DOX is simply immobilized on this interaction site and we can thus identify this site as the strongest one. However, this is only one such a strong interaction site which is available for a single DOX molecule. This is suggested by significant drop in pair energy when four molecules adsorb on iMp that is C4 case. Closer analysis of the nature of H-bonds in C3 case leads to the conclusion that they are formed with the nitrogen and oxygen O# as donors and oxygens in the phosphate backbone as acceptors. In the C4 case, i.e., when 4 DOX molecules are present the number of hydrogen bonds increases strongly up to 19 (or 4.75 per single DOX) and their nature also changes. In the C4 case, the most frequently observed H-bond form with O* atom as donor and oxygens from phosphate backbone as acceptors, other 4 the most abundant H-bonds uses the same sites of DOX as in the C3 case but the acceptor sites on the phosphate backbone are different. Thus, the strong site in the C3 case does not exist any more when more than one DOX molecule interacts with iMp.

Generally, DOX involves mainly these four interaction sites (N, O*, O#, and O@) as donors in the most frequently observed H-bonds in Figure 7. Other oxygen atoms actually do not take part in formation of hydrogen bonds. In cases of single DOX molecules, the H-bonds are mainly formed using oxygen atoms from phosphate backbones, other localizations are statistically negligible. On the other hand, in the cases of four DOX molecules also atoms belonging to nitrogenous bases appear as acceptors. In the C2 case atoms belonging to adenines appear with occupancy larger than 10% but these are only three hydrogen bonds. The other ones involve oxygens from phosphate backbones. In the case of the C4 system, the most abundant donor atoms from DOX are N, O*, and O# and they interact with acceptor atoms from iMp with strongly reduced representation of oxygen atoms from phosphate backbones. In the C4 case, we have only five H-bonds with occupancy bigger than 10% and only three of them involve oxygen atoms from phosphate backbones. In contrast, in the case of C2 the proportion was 13:3 thus in the case of unfolded iMu there is a larger availability of acceptor atoms from phosphate backbone.

### 2.5. Detachment of DOX from iM in Biased Calculations

In order to measure the binding strength of DOX in neutral and acidic pH we applied biased simulations. To that purpose, we performed steered detachments of DOX molecules from DOX-iM constructs C2 and C4 and measured work done during the detachments being the result of interaction between these two species. That work is directly related to the potential of mean force and is a good estimate of the free energy change accompanied the desorption of DOX from iM surfaces.

Technically, the steered detachments of DOX from iM were done using colvars [44] library linked to LAMMPS. The collective variable used to steer the dynamics was the distance between the center of mass of iM and the group of all DOX molecules. Thus, when the calculation was continuing the center of mass of DOX molecules was forced to follow (according to harmonic spring force) the moving point from the initial value to the target value with a constant velocity 1.95 Å ns^−1^. Therefore, at some point of time we observed detachment of one (or more) DOX molecules from the iM structure and this was accompanied by a jump in the work vs. distance plot. The height of that jump is directly related to the energy barrier against desorption or it is simply the binding free energy of that DOX molecule at a given site. Because the determined work usually depends on the instantaneous configuration of the system the binding free energy should be obtained by averaging many trajectories starting from different configurations and leading to the same or similar final configuration. However, in this case we found that more interesting will be analysis of individual trajectories because we can get insight into the stability of different states (adsorption sites) of DOX adsorbed on the iM. Moreover, the enforced detachment led to desorption of a single, two or even three DOX molecules simultaneously. Therefore, the final states differ significantly and without re-normalization of the results it would be difficult to draw reliable comparative conclusions. 

**Figure 8:**
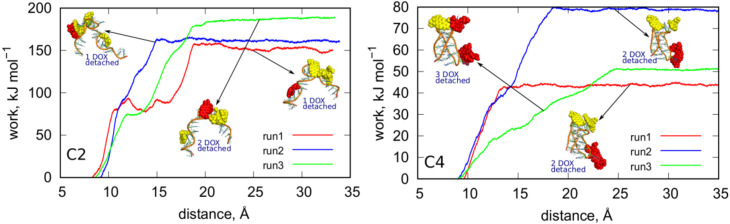
Works measured during the enforced detachment of DOX molecules from iM. Each curve (run1–run3) shows an independent trace started from a different initial state. These states are illustrated as the insets and are linked with the corresponding plots by arrows. The red DOX molecules are the molecules which detached as a result of the enforced detachment. The number next to a given inset indicates the number of DOX molecules which were simultaneously detached from the iM surface.

Figure 8 shows the determined plots of work vs. distance for C2 and C4 cases. The distances were computed between centers of mass of iM and all DOX molecules. Thus, the external force imposed on DOX spreads over all of them and it sometimes initially leads to migration of DOX on the surface of iM. This is the reason of the appearance of multiple steps on the plots (e.g. run1 in the C2 case). The plateau which appears on each curve at distances larger than 25 Å means that DOX (one or more molecules) was detached from the iM and diffuse in the bulk freely. Therefore, the height of the plateau is a measure of the total work necessary to desorb DOX from the iM.

Adsorption of DOX on the iMu is strong. The determined works in Figure 8 reach about 160 kJ mol^−1^ for detachment of a single DOX molecule. In run1 and run2 application of the steered dynamics led to detachment of a single DOX molecule but in run3 a cluster of 2 DOX molecules was desorbed. This cluster did not decompose in the bulk. In each case the DOX molecules wandered on the surface of iMu for some time before detachment. It is most visible in run1 where DOX molecule was walking for quite a long time (first plateau) until it finally approached the other DOX molecule and formed a structure similar to those from run2 and run3. It finally detached (second plateau) and this means that direct interaction of DOX with iMu is strong and it becomes weakened when a cluster of few DOX molecules is formed. Then, the detachment becomes facilitated over further migration on the iMu surface.

In the C4 case, the works are much lower and more diverse when changing an initial configuration from which the enforced detachment starts. Moreover, in this case DOX desorbs easily in clusters of two or even three molecules. The detected works change from ca. 45–80 kJ mol^−1^ depending on the starting configuration (run1–run3). However, it seems that adsorption of DOX on iMp surface is much weaker than in the case of iMu and DOX can desorb spontaneously due to thermal agitation only. Interestingly, in run3 we first observed migration of two DOX molecules from the bottom part of iMp to the top part where the other 2 DOX molecules resided. It is visible in the plot in Figure 8 which illustrates the increasing strain in the system until the DOX molecule finally escapes to the bulk and the plateau forms. Generally, in the C4 case DOX detach in groups of two or three molecules and this means that interaction between DOX molecules is comparable to the interaction with iMp. Our previous, more detailed studies, led to the conclusion that DOX exists in form of single molecules and clusters up to 4–5 molecules in water solution and these clusters change their size spontaneously by migration of DOX molecules between them. We previously determined the binding free energy of DOX in clusters and it was found that that binding free energy is about 29 kJ mol^−1^ [43] Thus, this is another proof of weak binding of DOX to iMp and ability of spontaneous release when iMu switch into iMp due to pH change.

## 3. Conclusions

In this work, we studied whether carbon nanotubes and iM molecules can act as pH sensitive carriers of doxorubicin. Two different concepts were tested, namely (i) making use of the iM as controllable caps of the nanotube ends due to its folding/unfolding property and (ii) using this same property of iM as intercalating factor of DOX. We found that the concept (i) is impossible to work properly since DOX molecules are not effectively locked in the CNT interior by the iMu molecules adsorbed on the CNT surface. Perhaps covalent binding of iMu to CNT tips would lead to the requested property but at this moment we focused only on the simplest architectures which do not involve sophisticated fabrication methods. On the other hand, we found that DOX and iMu molecules effectively intercalate each other and attach to the CNT strongly. This leads to obtaining of a needle-like transporter where DOX is strongly bound within the iMu/DOX phase and cannot escape to the bulk. At the same time, DOX molecules are less exposed to the solvent and this should reduce the side effects of DOX administration during the transportation stage taking place at physiological pH. Reduction of pH is associated with the folding of iMu to iMp structure and it is expected that that process should occur in the acidic microenvironment of tumor tissue. We found that after transition of iMu to iMp the binding of DOX is strongly weakened; thereafter, its mobility within the iMp/DOX phase increases significantly and finally it is able to escape to the bulk. Thus, the concept (ii) turns out to be feasible and CNT/iM/DOX constructs can act as pH controlled DOX delivery systems to tumor sites.

In publications where the formation of iM at acidic pH was the driving force for DOX release quite sophisticated mechanisms of that process were postulated [12,13,14,15,16,17]. As mentioned in the Introduction section DOX was assumed to be intercalated within the duplex formed by iMu harpin and a second complementary strand. The transition to the iMp form requires then a cleavage of Watson-Crick pairs in favor of formation of the Hoogsteen pairs. However, we found that the immobilization of DOX by iMu hairpins on the CNT surface at neutral pH is effective without the secondary DNA strand. Moreover, the release at acidic pH, when iMu switches into iMp, is a simple and thermodynamically feasible mechanism. Therefore, this mechanism should also be considered in the design of i-motif based carriers of DOX. 

## Figures and Tables

**Table 1:** Definitions of the analyzed systems.

System ID	Number of iM Molecules	CNT Chirality/Diameter, Å	Protonation State of Cytosines (P/N) *	Number of DOX Molecules
A	6	(20,0)/15.7	N	10
B1	6	(10,0)/7.8	N	0
B2	6	(10,0)/7.8	N	24
B3	6	(10,0)/7.8	P	0
B4	6	(10,0)/7.8	P	24
C1	1	-	N	1
C2	1	-	N	4
C3	1	-	P	1
C4	1	-	P	4

* P—protonated cytosines; N—nonprotonated cytosines.

**Table 2:** Mean pair interaction energies between various compounds determined from the last 10.8 ns of the simulations. The energies are given in kJ mol^−1^. The column ‘Clusters’ informs about the number of clusters formed by iM molecules (fCNT and DOX molecules are not included in cluster analysis). SASA is the solvent accessible surface area calculated for DOX molecules.

System	iM- iM	iM- CNT *	iM- fCNT #	iM- DOX	DOX- CNT *	DOX- fCNT #	DOX- DOX	Clusters	SASA, Å^2^
B1	14790	−1632	−2048	-	-	-	-	2	-
B2	15929	−805	−858	−7887	−1071	−1174	23905	1	6792
B3	9093	−896	−1158	-	-	-	-	4–5	-
B4	1239	−467	−552	−7000	−733	−858	24059	4	9432

* Nanotube without functional groups; # Nanotube with functional groups.

**Table 3:** Mean pair interaction energies between various systems components. The energies are calculated per single DOX molecule. The column H-bonds informs about the number of hydrogen bonds observed between DOX and iM during the last 10.8 ns of calculations (in parentheses the number of H-bonds per single DOX molecule is shown).

System	DOX-iM, kJ mol−1	iM-Water, kJ mol−1	DOX-Water, kJ mol−1	H-Bonds
C1	−252	−11131	−445	15
C2	−440	−9740	−272	34 (8.5)
C3	−475	−9334	−300	3
C4	−204	−9262	−444	19 (4.75)

## Abbreviations

CNT	Carbon nanotube
fCNT	Functionalized carbon nanotube
iM	Cytosine rich telomeric DNA sequence able to fold into i-motif
iMu	iM with nonprotonated cytosines
iMp	iM with protonated cytosines
DOX	Doxorubicin
